# Enhanced Digital Gradient Sensing Using Backlight Digital Speckle Target

**DOI:** 10.3390/s20226557

**Published:** 2020-11-17

**Authors:** Baofei Fu, Chenzhuo Li, Bo Dong, Pan Ou

**Affiliations:** 1 School of Instrumentation Science and Opto-electronics Engineering, Beihang University, Beijing 100191, China; fubaofei@buaa.edu.cn (B.F.); oupan@buaa.edu.cn (P.O.); 2 Institute of Solid Mechanics, Beihang University, Beijing 100191, China; cz.li@buaa.edu.cn; 3 School of Automation, Guangdong University of Technology, Guangzhou 510006, China

**Keywords:** digital gradient sensing, backlight illumination, angular deflection, stress gradient

## Abstract

Digital gradient sensing (DGS) is a non-contact and full-field optical measurement technique, which assesses mechanical behaviors of transparent materials or specular structures by measuring angular deflections of light rays. However, owing to the poor light-gathering capability of its imaging system, the dynamic performance of DGS is heavily restricted. Here, a method of enhancing the dynamic performance of DGS by improving its speckle target is proposed. The method employs the technique of backlight illumination, which significantly increases the utilization efficiency of light, shortens the exposure time, and enhances the dynamic performance of DGS. Additionally, it also uses the optimized digital speckle pattern to improve the measurement precision and accuracy. For validation, a comparison experiment was conducted, proving that the proposed method can improve the utilization efficiency of light by about 80 times and improve the quality of the speckle images by about 40%. Real tests, including a uniaxial tension test using transmission-mode DGS (t-DGS) and a three-point bending test using reflection-mode DGS (r-DGS), were also carried out, showing the efficacy and high compatibility of the proposed backlight digital speckle target. In summary, this simple method greatly improves the performance of DGS, which can be used as a standard method in both t-DGS and r-DGS.

## 1. Introduction

Digital gradient sensing (DGS), originally developed in 2012 by Tippur et al. [1], is a digital image correlation (DIC)-based non-contact and full-field optical technique, which assesses mechanical behaviors of transparent materials or specular structures by measuring angular deflections of light rays. DGS can be classified as transmission-mode DGS (t-DGS) and reflection-mode DGS (r-DGS), where the former one measures non-uniform stress distribution induced angular deflections to characterize stress gradient fields of transparent materials, while the later one measures angular deflections reflected off an optically reflective surface to estimate the surface slopes of specular structures [1,2]. Due to its advantages of simple implementation, high accuracy, and high computational efficiency [3,4,5], DGS has been successfully applied in many fields—e.g., material testing, fracture mechanics, impact dynamics, and high-temperature characterizations—showing great potential in recent studies [5,6,7,8,9,10,11,12,13,14].

During a DGS measurement, the images of the speckle target before and after specimen deformation are firstly acquired by an imaging system, then the angular deflections of light rays can be estimated from the acquired images using the established physical model and DIC algorithm. Since the angular deflections are associated with the stress distributions of transparent materials (*t*-DGS) or surface slopes of specular structures (*r-*DGS), the mechanical behavior of the specimen can be assessed. Therefore, the measurement resolution and accuracy of DGS are highly determined by the quality of acquired speckle images. In practical measurements, there are two aspects that influence the quality of acquired speckle images—i.e., speckle target and imaging system. Currently, the speckle target is fabricated by spraying black and white paints onto a planar plate and illuminated by using external light sources; the imaging system is composed of a digital camera and a telephoto lens with small aperture. Since the imaging system of DGS has a large focal length and a small aperture size, the light-gathering capability of it is very poor. As a result, only a small portion of reflected light can be gathered from the illuminated speckle target by the imaging system. In practical uses, the poor light-gathering capability restricts the exposure time, also the acquisition rate, which means DGS cannot work well in high-speed measurements—e.g., fractures and shocks.

To improve the dynamic capability of DGS, some studies employ ultra-high-power light sources, instead of the regular ones, to shorten the exposure time. For example, Miao et al. replaced the ordinary white light source with a high-power white LED cold light source to illuminate the speckled target, when studying the surface morphology of silicon wafer and the stress gradient of crack tip [15]. Periasmy et al. used two high-energy strobe lights to collect high-quality speckle images and quantitatively analyzed the stress gradient near the crack and at the impact edge in the experiments of cracks and punching in PMMA plates [7]; Dondeti et al. utilized a wide-spectrum Xenon flash lamp to illuminate the speck target in the study of the dynamic fracture process of sodium calcium glass [16,17]. Though the employment of ultra-high-power light source can improve the dynamic capability of DGS, it requires extremely precise synchronization between the light sources and the high-speed camera, which greatly increases the difficulty of DGS implementation. In addition, since the light-gathering capability of DGS is very poor, only employing a high-power light source cannot fully meet the requirements of high-speed applications. Therefore, a method that can improve the utilization efficiency of light in DGS measurement is highly required. Very recently, Tippur et al. proposed a simplified *r*-DGS to solve the problem of low utilization efficiency of light. It abandons the beam splitter in the classic *r*-DGS device and directly reflects the speckles into the camera through the specular surface of the specimen at a known angle (usually 45°) for imaging [18]. The method improves the utilization efficiency of light by about four times, but it makes the calculation procedure more complex. More importantly, the idea is only applicable for *r*-DGS, showing certain limitations in practical implementations. Similar to the experimental setup of simplified *r*-DGS, deflectometry for specular surfaces usually adopts LCD screens to display speckle pattern as an information carrier [19,20]. However, to apply this technique in DGS, the low spatial resolution of LCD screens limits the resolution of the displayed speckle pattern, making it hard to effectively measure the strain concentration area—e.g., near the tip of notches. Additionally, the insufficient brightness of LCD screens makes it unrealistic in dynamic measurement.

In this work, a method of enhancing the utilization efficiency of light in DGS by improving its speckle target is proposed. The method combines the backlight illumination and digital speckle pattern, which can be applied in both *t*-DGS and *r*-DGS. First, this backlight digital speckle target utilizes the background light rays transmitted through the transparent/opaque speckles to form speckle patterns on the camera sensor, which can significantly enhance the light utilization efficiency (for about 1~2 orders of magnitude) and increase the speckle image contrast. Second, the proposed speckle target uses a computer-generated digital speckle pattern, which can increase the measurement resolution and accuracy by optimizing the speckle size, density, and distribution. In the remainder of this manuscript, the principles of DGS and backlight digital speckle target are firstly introduced. Then, validation experiments are carried out to validate the effectiveness of the proposed method. Finally, real tests—i.e., a uniaxial tension test using the enhanced *t*-DGS and a three-point bending test using the enhanced *r*-DGS—are performed to verify the applicability of the method.

## 2. Methodology

### 2.1. Digital Gradient Sensing

**Figure 1:**
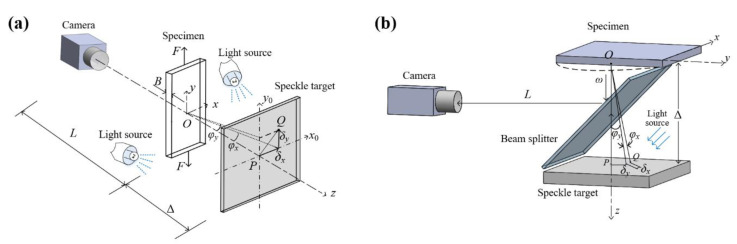
Schematics of (**a**) transmission-mode DGS, (**b**) reflection-mode DGS.

The schematics of *t*-DGS and *r*-DGS are shown in Figure 1. The system of *t*-DGS, shown in Figure 1a, is composed of a digital camera, a speckle target, and two symmetrically placed external light sources. Before the measurement, the transparent specimen is placed in front of and parallel to the speckle target at a known distance Δ. The digital camera is placed in front of the specimen at a large distance *L* (>>Δ) with its optical axis perpendicular to the specimen surface. During the measurement, the thickness and refractive index of the specimen change with the external loading because of the photoelastic effect, which deviates the transmitted light from its original propagation path and leads to the deformation of the speckle image acquired by the camera. By analyzing the speckle images captured before and after the deformation with the assist of DIC, full-field displacements of the speckle images can be firstly obtained. After considering the geometry of the *t*-DGS system, the angular deflections of light can be subsequently estimated, which can be used to conclude the full-field stress gradient of the specimen according to the photoelastic effect characteristic of the specimen. The relationship between the angular deflection and stress gradient [1] is

(1) $$\phi_{x;y}\approx \frac{\delta_{x;y}}{\Delta }=C_{\sigma }B\frac{\partial (\sigma_{xx}+\sigma_{yy})}{\partial x;y},$$

where *ϕ_x_*_;*y*_ represents the angular deflection in *x*- or *y*-direction, *δ_x_*_;*y*_ is the displacement component in *x*- or *y*-direction, *C_σ_* is the elasto-optic constant of the specimen, *B* is the initial thickness of the specimen, *∂*(*σ_xx_*+ *σ_yy_*)/*∂_x_*_;*y*_ represents the stress gradient in *x*- or *y*-direction.

The system of *r*-DGS, shown in Figure 1b, is composed of a digital camera, a beam splitter, a speckle target, and external light sources. The implementation procedure and measurement principle of *r*-DGS are similar to but different from *t*-DGS. Before the measurement, the specular surface specimen is placed parallel to the speckle target with a known distance Δ. Between them, the beam splitter is placed at an angle of 45° to the optical axis of the camera. During the measurement, the speckle images before and after deformation are firstly recorded by the camera. Then, the full-field displacement can be obtained by using the DIC algorithm. After that, the angular deflections of light caused by out-of-plane displacements of the specular specimen and its slope fields can be subsequently calculated based on the system geometry. The relationship between the angular deflection and surface slope [2] is

(2) $$\frac{\partial w}{\partial x;y}\approx \frac{1}{2}\phi_{x;y}\approx \frac{1}{2}\frac{\delta_{x;y}}{\Delta },$$

where *w* is the out-of-plane displacement of the specimen, *∂w/∂x;y* is the orthogonal surface slope.

Known from Equations (1) and (2), the measurement sensitivities of both *t*-DGS and *r*-DGS are determined by displacement *δ_x_*_;*y*_ and distance Δ. Since the sensitivity of *δ_x_*_;*y*_ is associated with the DIC algorithm and the imaging resolution, the state of the art DIC algorithm and a small field of view (FOV) imaging system are needed in DGS measurement. In addition, the distance Δ is related to the depth of field (DOF) of the imaging system—i.e., a large Δ requires a large DOF. Therefore, considering both the requirements, the imaging system of DGS needs to be adopted with a telephoto lens—i.e., a lens with large focal length and small aperture size. However, this in turn limits the light-gathering capability of the imaging system, resulting in the poor exposure time and sampling rate. As a result, the dynamic capability of current DGS is insufficient to meet the requirement of high-speed measurements.

### 2.2. Backlight Digital Speckle Target

**Figure 2:**
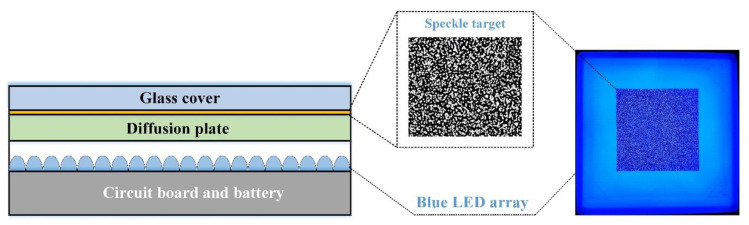
The schematic (left) and photograph (right) of the backlight digital speckle target. It consists of 5 layers—i.e., battery and circuit board layer (wireless power supply), blue LED array layer (high illuminance panel light source), diffusion plate layer (backlight homogenization), digital speckle pattern layer (blocking/transmitting backlight for high-contrast speckle pattern formation), and glass cover layer (protection).

To improve the dynamic capability of DGS, the most fundamental way is enhancing its utilization efficiency of light. In this paper, a backlight digital speckle target based on the combined use of backlight illumination and digital speckle pattern is proposed as shown in Figure 2. The proposed speckle target consists of 5 layers—i.e., battery and circuit board layer, blue LED array layer, diffusion plate layer, digital speckle pattern layer, and optical glass cover layer. The high capacity battery pack and circuit board are designed on the bottom layer, serving as wireless power supply. The blue LED array layer is a panel light source made of 20 × 20 blue LED bulbs, for emitting high illuminance blue light. The diffusion plate layer is made of a white diffusion plastic plate, which is used to homogenize the passing blue backlight. The digital speckle pattern layer is fabricated by a high-precision photoplotter, and the speckle pattern on it is designed by numerical simulation [21]. Note that even though digital speckle patterns can only be applied in DIC with a special method—e.g., water transfer printing [22]—it is very convenient to be used in DGS, because the speckle target in DGS is non-contact and reusable. On the top layer, the optical glass cover is used for protection.

During DGS measurement, the battery and the circuit board drive the blue LED array to emit high-intensity evenly distributed blue light, which then homogenized by the diffusion plate to become uniform backlight. The uniform blue backlight then passes through the transparent/opaque texture of the digital speckle layer and forms high contrast speckle images on the camera sensor, which can be used for full-field displacement tracking and DGS analysis. It is noteworthy that the use of blue light is inspired by the idea of active imaging [23], and therefore, a blue bandpass filter is equipped in front of the lens to enhance the system robustness against the ambient illumination variation.

Comparing with the conventional speckle target, this backlight digital speckle target features some extraordinary advantages in practical uses:

(1) High dynamic measurement capability. The backlight speckle target selectively shades the blue backlight through the transparent/opaque texture of digital speckle layer, and allows the light emanating from the target to be captured by the imaging system directly, which can improve the utilization of light source, shorten the exposure time, and enhance the high-speed measurement performance of the system.
(2) High measurement accuracy. The measurement accuracy of DGS is associated with the speckle size, speckle distribution, and speckle contrast. The proposed backlight digital speckle target utilizes the Gaussian speckle pattern simulation algorithm to generate the digital speckle pattern whose speckle size, density, and distribution can be designed to better match the real measurement scenario (e.g., the size of the specimen and the FOV). Therefore, the measurement accuracy of DGS can be improved after employing the digital speckle target [24]. In addition, the employment of backlight illumination can enhance the speckle contrast of the acquired speckle images [25]. The optimized speckle quality and contrast promote the accuracy of DIC point-wise tracking of displacement, and therefore, improves the DGS measurement accuracy.
(3) High system integration and better operation. In conventional DGS methods, the speckle target and the lighting system are two separated parts. Before the measurement, the angle of the light source needs to be cumbersomely adjusted by the operator, so as to achieve the uniform illumination of the speckle target. The proposed backlight speckle target not only combines the illumination source and the speckle target, improving the system integration, but also effectively solves the tedious light source adjustment problem.
(4) Strong robustness against the ambient light variation. The backlight speckle target adopts the blue backlight source and the corresponding blue bandpass filter to realize the blue light active imaging conveniently, which is expected to suppress the light variation in the ambient environment, and improve the stability and reliability of the measurement system [23].
(5) High system compatibility. Compared with the simplified *r*-DGS method proposed by Tippur et al. [18], the backlight speckle target solution has higher system compatibility. That is, compatible with not only the conventional *r*-DGS but also the conventional *t*-DGS and the simplified *r*-DGS. The high compatibility greatly promotes the proposed speckle target to improve the light source utilization and integration of all DGS systems.

In addition, it should be noted that such a backlight speckle target can also be realized by a tablet computer display [26], but compared with the above-mentioned specially fabricated speckle target, low speckle resolution problems may exist. The print resolution of the used photoplotter is generally up to 1 μm, while the dot pitch of the display equipped on the tablets is generally poorer than 50 μm. Considering that in DGS measurement, the measurement FOV is usually small and the measurement accuracy is required to be high, so the use of digital speckle based on tablet display will suffer great limitations.

## 3. Experiments

### 3.1. Validation Experiment

**Figure 3:**
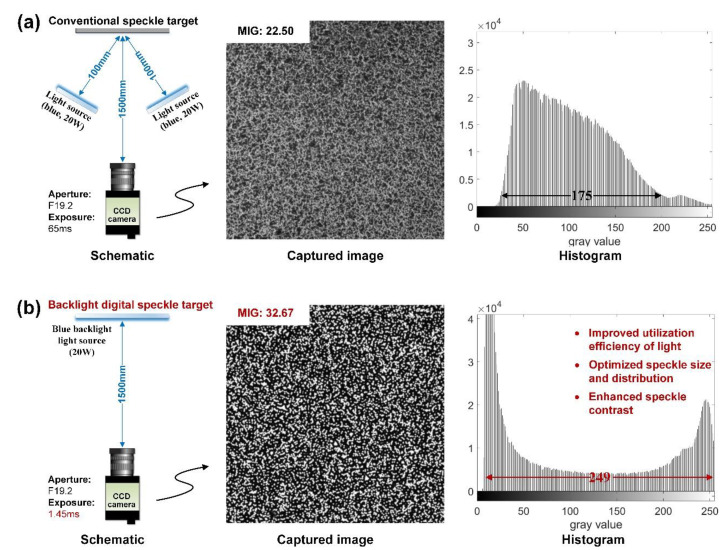
Experimental setups and captured images: (**a**) conventional speckle target; (**b**) backlight digital speckle target.

To verify the effectiveness of the proposed method, an experiment of comparing two different speckle targets—i.e., the conventional speckle target and the backlight digital speckle target—was carried out. As shown in Figure 3, the speckle target was placed in front of a camera (GS3-U391S6M-C, active pixels: 3376 × 2704 pixels, FLIR, Germany; lens: TAMRON AF 70–300 mm F/4-5.6 Di LD 1:2 MACRO, Model A17, aperture size: F19.2) by 1500 mm (similar to the working distance in the real measurement of DGS) and adjusted perpendicular to the optical axis of the camera. As shown in Figure 3a, the conventional speckle target was made by spraying black/white matte paint, and two blue LED panels (power: 20 W) were used for illumination with a distance 100 mm to the target. The target diffusely reflects the incident light to the camera and to form speckle images. As shown in Figure 3b, the backlight speckle target used one of same blue LED panels as the backlight source. Passing through the digital speckle layer with transparent/opaque texture, the emanating light directly enters the camera to form a speckle pattern. It is worth noting that the digital speckle layer was numerically generated based on Gaussian simulation algorithm (4000 × 4000 pixels pattern size, 3–6 pixels speckle size and 10% speckle density) and fabricated by high-precision photoplotter. During the experiment, speckle images of the conventional speckle target and backlight speckle target with normal gray distribution (i.e., no over-exposure and over-darkness) under different aperture values (i.e., F19.2, F9.6, F6.4, and F4.8) were captured by adjusting the exposure time. The different exposure time was used to evaluate the light source utilization of the two speckle targets. Furthermore, the quality of the speckle images was analyzed and assessed by mean intensity gradient (MIG).

**Figure 4:**
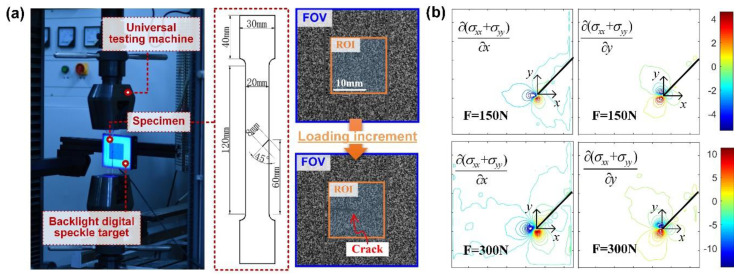
Experimental setup of enhanced *t*-DGS and its measurement results. (**a**) Experimental setup and captured speckle images; (**b**) measured stress gradient contours at tensile load of 150 and 300 N.

The conventional speckle image and backlight speckle image acquired under the smallest aperture and their gray distribution histogram are shown in Figure 4. With the increase in aperture, the exposure time required to maintain the normal grayscale distribution are listed in Table 1. As expected, the exposure time of both speckle images increases continuously with the decrease in aperture (i.e., the increase in F number). It also can be seen from Table 1 that under the same aperture, the exposure time of the conventional speckle image is about 40 times longer than that of the backlight speckle image. Considering that only one of the same LED panels was used in the backlight speckle target, it can be concluded that the backlight speckle target increases the light source utilization by about 80 times, which can greatly enhance the dynamic measurement performance of DGS and promote its application potential in high-speed measurement (such as crack propagation and impact test).

It can be seen from Figure 3 intuitively that the digital speckle image has higher contrast and wider gray distribution than the conventional speckle image. To quantitatively exhibit the advancement of the proposed digital speckle target in improving the quality of the speckle images, the MIG of both speckle images under different aperture setting was evaluated and compared, as shown in Table 2. As a global speckle quality assessment parameter, MIG was first proposed by Pan et al. [24], which evaluates the speckle image quality through its global average intensity gradient. Therefore, the higher, the better. Comparing the calculated MIG of the backlight digital speckle image with that of the conventional speckle image, it can be seen that under the same aperture size, the former was about 50% higher than the latter, which is consistent with the improvement in optimized digital speckle patterns for DIC reported in the literature [27]. Hence, the high speckle contrast and high speckle quality offered by the proposed backlight digital speckle target are verified.

### 3.2. Stress Gradient Field Measurement Using the Enhanced t-DGS

To verify the feasibility of the proposed backlight speckle target in *t*-DGS tests, the stress gradient distribution near the crack tip of a polymethyl methacrylate (PMMA) planar specimen with a 45° notch was measured in a uniaxial tensile test. The experimental setup is shown in Figure 4a. The PMMA specimen was made by laser cutting, and its dimension is shown in the figure, where the width of the test section was 20 mm and the thickness was 4.8 mm. At the middle of the test section, a 45° crack with a length of 8 mm was prefabricated by laser cutting (width: 500 μm). Before the experiment, the specimen was clamped by the grips of the universal testing machine (UTM). The backlight speckle target was placed behind the specimen at Δ = 35 mm, and the imaging system (used in the verification experiment) was placed in front of the specimen at a distance of *L* ~ 1490 mm. To ensure a proper FOV size and a sufficient depth of field, the focal length of the zoom lens was adjusted to *f* = 180 mm, and the aperture was set as the smallest (F19.2). After adjusting the lens to focusing on the speckle target, a reference speckle image was captured through the undeformed specimen. Then, the specimen was loaded by the UTM in displacement at 0.3 mm/min and the deformed speckle images were simultaneously collected by the camera at 9 frames per second (FPS), until the fracture of the specimen. After collecting the reference speckle image and all the deformed speckle images, the *x*- and *y*-directional displacement field near the crack tip of the specimen can be obtained by using 2D-DIC analysis [28,29]. To conduct 2D-DIC analysis, a region of interest (ROI) was first specified on the reference image near the crack tip of the specimen, as shown in Figure 4a. By specifying a grid step size (5 pixels), the ROI was divided into evenly distributed calculation points. At each calculation point, a 51 × 51 pixels subset was selected to represent the location of the center calculation point. By comparing the similarity of the subset before and after deformation according to the ZNSSD correlation function, the sus-pixel displacement of the subset can be iteratively calculated using the IC-GN algorithm. Applying this optimization process for all calculation points in the ROI, the full-field displacement components *δx* and *δy* of the speckle images caused by the deformation of the specimen at different stages can be obtained, and the angular deflection of light *Φ_x;y_* can be obtained from the system geometry. Therefore, the full-field stress gradients of the specimen during the deformation process can be derived based on Equation (1) and the elasto-optic constant of the specimen material. The calculated stress gradient contours at tensile load of 150 and 300 N are shown in Figure 4b, where the thick black solid line represents the prefabricated crack. It can be seen that the stress gradient field in the *x* direction is approximately antisymmetric along the crack tip, while the stress gradient field in the *y* direction shows a strong stress gradient variation at the end and the both sides of the crack tip. This experimental phenomenon is consistent with the result reported in the literature [14] of a similar experiment using conventional *t*-DGS (where the detailed descriptions and result analysis can be found). Therefore, it is verified that the proposed method can be easily applied to the regular *t*-DGS experimental setup. The feasibility of the proposed backlight digital speckle target in *t*-DGS tests is also verified.

### 3.3. Plane Slope Measurement Using the Enhanced r-DGS

**Figure 5:**
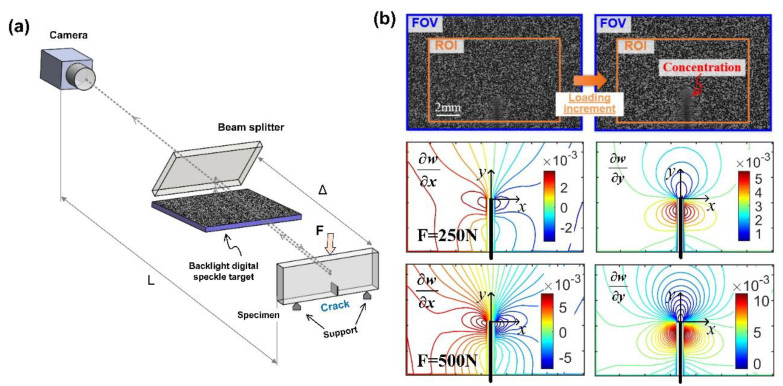
Experimental setup of enhanced *r*-DGS and its measurement results. (**a**) Experimental setup; (**b**) measured slope fields under uniaxial load of 250 and 500 N.

As mentioned in Section 2.2, one of the extraordinary advantages that the proposed backlight digital speckle target can offer is its high compatibility for all DGS systems. To verify this, the slope fields of a single edge notched PMMA specimen in the three-point bending experiment were measured using the *r*-DGS. The experimental setup is shown in Figure 5a, where the size of the specimen coated with reflective film on the surface was 150 × 50 × 8 mm. A 10 mm vertical crack was prefabricated from the middle of one long side by laser cutting (width: 500 μm). This side of the specimen was supported by specially designed fixtures with a spacing of 100 mm and placed on the lower crosshead of the UTM. In front of it, the beam splitter was placed at 45° with the separation distance Δ = 45 mm, and the backlight digital speckle target was placed horizontally below it. The image acquisition device the same as in the *t*-DGS experiment was placed perpendicularly to the specimen at the distance of *L* = 145 cm and all the parameter settings kept consistent. At the start of the experiment, a speckle image reflected from the specular surface of the specimen and transmitting through the beam splitter was captured by the camera as the reference image. Then the specimen was compressed to 500 N by step loading of 50 N. At every load stage, the speckle image reflected from the deformed specimen was captured. All the reference speckle images and deformed speckle images were analyzed by the 2D-DIC algorithm used in the *t*-DGS to obtain the full-field displacement. Therefore, the *x*- and *y*-directional slope fields can be calculated according to Equation (2) with the known system geometry. The calculated *x*- and *y*-directional slope fields near the crack tip at the compression load F = 250 N and F = 500 N are shown in Figure 5b, where the black vertical line represents the prefabricated crack. It can be seen that the *x*-directional slope field exhibits an antisymmetric distribution with respect to prefabricated cracks, and *y*-directional the slope field shows a symmetric distribution with respect to prefabricated cracks, which is consistent with the result of three-point bending experiment reported in the literature [18] using conventional *r*-DGS (where the detailed descriptions and result analysis can be found). Therefore, the feasibility of the proposed backlight digital speckle target in *r*-DGS tests is verified. Considering the effectiveness of the proposed method in *t*-DGS has been proved in Section 3.2, it can be concluded that the proposed method is highly compatible in both *r*-DGS and *t*-DGS measuring systems, without changing the physical model of the measuring system or complicating its implementation.

## 4. Conclusions

A backlight digital speckle target is proposed to enhance the performance and practicability of DGS. First, the method employs the backlight illumination technique, which increases the utilization efficiency of light, shortens the exposure time, and enhances the dynamic capability of DGS. Second, it uses the digital speckle pattern to improve the quality of speckle images, which can enhance the measurement precision and accuracy. In addition, the combination of backlight illumination and digital speckle pattern also simplifies the operation and has high integration and compatibility. For validation, a comparison experiment was firstly conducted, which proves that the proposed speckle target can improve the utilization efficiency of light by about 80 times and enhance the quality of the speckle images by about 40%. Furthermore, application experiments of a uniaxial tension test using *t*-DGS and a three-point bending test using *r*-DGS were also carried out, proving the feasibility and high compatibility of the proposed backlight speckle target. In summary, this simple method greatly improves the performance of DGS, which can be used as a standard method in DGS technologies.

In our next work, a special technique [30], which is capable of measuring specimen deformation while not influencing the light transmission of transparent materials, will be employed to assist *t*-DGS measurement for achieving simultaneous measurement of in-plane deformation and stress gradient field.

## Figures and Tables

**Table 1:** Exposure time of different aperture sizes and different lighting methods.

Aperture Size	Exposure Time (Conventional Method)	Exposure Time (Backlight Method)	Comparison
F19.2 (small)	65,000 us	1450 μs	44.8 times decreased
F9.6 (medium)	13,500 us	380 μs	35.5 times decreased
F6.4 (medium)	8300 us	220 μs	37.7 times decreased
F4.8 (large)	5000 us	125 μs	40.0 times decreased

**Table 2:** Mean intensity gradient (MIG) of two speckles with different aperture sizes.

Aperture Size	MIG (Conventional Method)	MIG (Backlight Method)	Comparison
F19.2 (small)	22.50	32.67	45.2% improved
F9.6 (medium)	25.93	33.51	29.2% improved
F6.4 (medium)	24.81	34.43	38.7% improved
F4.8 (full)	24.34	35.86	47.3% improved
